# Managing Mental Health Crises in Indonesian Universities: A Reflexive Thematic Analysis of Psychologists' Experiences

**DOI:** 10.2174/0117450179452308260709213331

**Published:** 2026-07-13

**Authors:** Aisha Sekar Lazuardini Rachmanie, Yesica Grahita Rumanti Mahambara, Restu Tri Handoyo, Subandi

**Affiliations:** 1 Faculty of Psychology, Universitas Gadjah Mada, Yogyakarta, Indonesia

**Keywords:** Crisis Intervention, Higher Education, Indonesia, Mental Health Services, Psychologists, Qualitative Research, Students

## Abstract

**Introduction:**

Mental health issues have become a major concern in universities worldwide, including in Indonesia. Various mental health challenges experienced by students can trigger crises, such as urges to self-harm or even suicidal ideation. Such crises require immediate intervention. However, access to mental health emergency services remains limited and lacks standardized guidelines. This study aims to explore participants' experiences in handling mental health emergencies in university settings, with the hope of providing insights for the development of standardized mental health emergency service guidelines.

**Methods:**

Data collection was conducted through focus group discussions involving six psychologists working in university-based mental health practices. The collected data were analyzed using reflective thematic analysis.

**Results:**

Through reflective thematic analysis, four primary themes emerged, namely the understanding and interpreting crisis; navigating crisis assessment in complex situation; professional responses in crisis intervention; and challenges in managing crises.

**Discussion:**

The findings of this study reveal the complexity of understanding and managing psychological crisis cases, including significant challenges in doing so effectively. These findings are consistent with previous research on psychological services and provide insights into how to manage clients experiencing crises. These insights make a valuable contribution to the development of regulations and the implementation of standardized mental health crisis services in higher education institutions.

**Conclusion:**

Overall, this study demonstrates that crisis management in university mental health services involves diverse understandings of crisis, varied intervention practices, and multiple practical challenges. These findings highlight the importance of developing clearer frameworks to support mental health emergency services in higher education.

## 1 INTRODUCTION

Mental health has become a primary concern for universities worldwide. A survey of 14,000 first-year students across 19 higher education institutions in eight countries found that 35% of students had at least one mental health disorder, primarily major depressive disorder (21.2%) and generalized anxiety disorder (18.6%) [1]. This trend is mirrored in Indonesia, where screening via the Self-Report Questionnaire (SRQ-20) identified psychological distress in 76.9% of students [2]. Another study reported that 95% of students experienced anxiety disorders [3]. Similarly, 86.8% of participating students experienced high levels of anxiety [4]. In addition to anxiety, mental health problems are also reflected in the presence of depression and suicidal ideation [3, 4].

These mental health challenges occur during emerging adulthood. During this developmental phase, individuals navigate complex transitions across social and emotional domains [3, 5]. When individuals lack adaptive coping strategies, these stressors may lead to maladaptive responses [2, 3, 6]. These risks are compounded by low mental health literacy and persistent societal stigma, which discourage professional help-seeking and leave students vulnerable to acute psychological crises [7, 8].

Psychological crises occur when individuals encounter stressful situations that exceed their coping capacity, disrupting their psychological equilibrium and leading to intense emotional distress [9]. Among university students, such crises may be triggered by multiple stressors, including academic pressure [10], interpersonal conflicts [11], financial difficulties [12], and challenges related to identity development [13]. These factors can contribute to emotional dysregulation, particularly when coping resources and social support are insufficient. Because many mental disorders first emerge during emerging adulthood [14], exposure to significant stressors during this stage may increase vulnerability to crisis states, including severe psychological distress, self-harm behaviors, and suicidal ideation.

Given the potential for such emergencies, it is essential to establish emergency mental health services. These services should be specifically designed to respond to the needs of individuals experiencing psychological crises in a timely, effective, and confidential manner. Such services may include psychological first aid, stabilization of the individual’s condition, symptom mitigation, and pharmacological intervention when necessary [15-17]. While some Indonesian hospitals and universities have implemented mental health emergency services, their effectiveness is limited by narrow geographic coverage and a lack of standardized operational guidelines. Currently, Indonesia lacks national protocols for mental health emergencies, contrasting with established frameworks in the United States, Australia, and the United Kingdom [18-22].

The policy landscape in Indonesia remains largely reactive. The 2014 Mental Health Law lacks widespread regional implementation, leaving many universities without formal crisis response protocols or trained personnel [23, 24]. Systemic constraints further exacerbate this issue; with only 0.31 psychiatrists and 2.52 mental health nurses per 100,000 population, Indonesia falls significantly below WHO recommendations [25]. Such systemic constraints underscore the urgency of developing university-based intervention models, particularly in rural or underserved areas.

Given these issues, there is an urgent need for culturally adapted, evidence-based frameworks that guide university psychologists in managing mental health crises. While existing literature highlights the prevalence of student mental health problems and systemic barriers, limited research has examined how psychologists actually assess and manage crises within university settings, particularly in the Indonesian context. This study addresses this gap by exploring psychologists’ lived experiences in managing mental health emergency cases in Indonesian universities. By analyzing their strategies and the challenges encountered, this research provides practice-based insights to inform the development of structured crisis response frameworks in higher education. To achieve this objective, the study formulates two research questions: (1) How do psychologists experience the management of mental health emergency cases? and (2) What steps are necessary in handling mental health emergency cases?

## 2 METHODS

### 2.1 Study Design

This qualitative study utilized experiential–clinical focus group discussions (FGDs) [26] to explore psychologists’ experiences in managing university-based mental health emergencies. FGDs were selected because the method allows participants with similar professional backgrounds to collectively reflect on professional experiences, enabling insights to emerge through interaction, comparison of perspectives, and shared reflection. Such interaction can help elicit experiential and practice-based knowledge that may not emerge as readily in individual interviews [27].

Data adequacy was determined using the concept of information power [28]. Given the study's specific aim, the specialized expertise of the participants, and the high quality of professional dialogue, a sample of six psychologists was deemed sufficient to provide rich, meaningful insights. During the session, participants were divided into two smaller groups consisting of three psychologists each. This format was used to facilitate more focused interaction and to ensure that each participant had sufficient opportunity to elaborate on professional experiences. For discussions involving participants with specialized expertise and complex professional practices, smaller groups can support deeper reflection and more detailed exchanges while still maintaining interactive dialogue among participants.

### 2.2 Participants

Participants included six psychologists (five women, one man; aged 35–50) affiliated with university mental health emergency services. Inclusion required direct involvement in these services, while those with less than one year of crisis management experience were excluded. To ensure depth and interactive quality, participants were divided into two groups of three. This smaller format supports detailed exchanges and deeper reflection, which is particularly effective for complex professional topics [26].

### 2.3 Procedure

This study received ethical approval from the Ethics Committee of the Faculty of Psychology, Universitas Gadjah Mada, Indonesia (Approval No. 5878/UN1/ FPSi.1.3/SD/PT.01.04/2024). Prior to data collection, the researchers prepared an FGD guideline and an informed consent form to be provided to participants. Participants were selected purposively from psychologists working at the University Mental Health Emergency Service who met the inclusion criteria.

The 120-minute offline session was led by a licensed psychologist as facilitator, supported by an observer and a note-taker. After obtaining informed consent and explaining the research objectives, the facilitator guided the discussion based on semi-structured guidelines. The session concluded with a summary by the note-taker, and all discussions were audio-recorded for accurate transcription. Following data collection, the researchers conducted data analysis and prepared the research findings for inclusion in the manuscript.

### 2.4 Data Analysis

The collected data were analyzed using reflexive thematic analysis [29]. This approach was used to identify patterns of meaning. The process involved: (1) data familiarization through repeated reading, (2) inductive coding of narratives, (3) theme generation, (4) refinement against the dataset, and (5) defining and naming themes. The interview transcripts were coded manually using a spreadsheet in Google Sheets (Google LLC, United States) to organize codes and emerging themes.

Consistent with the reflexive thematic approach, the research team acknowledged their positionality as psychologists familiar with mental health practice, recognizing that this background influenced data interpretation. Rigor was maintained through continuous reflexive sessions to examine how professional assumptions shaped coding and theme development. These discussions facilitated a deeper interpretative engagement, ensuring the thematic structure was critically refined throughout the analytic process. To enhance trustworthiness, member checking was performed [30], where participants reviewed both the transcripts for accuracy and the final themes for interpretative validity.

## 3 RESULT

The focus group discussion (FGD) identified four primary themes regarding university-based emergency mental health services: (1) understanding and interpreting crisis, (2) navigating crisis assessment in complex situations, (3) professional responses in intervention, and (4) challenges in managing management challenges. These themes and their respective subthemes are summarized in Table **1**.

### 3.1 Understanding and Interpreting Crisis Situation

#### 3.1.1 Acute Crisis Presentations Requiring Immediate Responses

Crises encountered in the emergency service often involve acute psychological distress requiring immediate response. Participants described that many clients contacted the emergency hotline during late-night hours, requiring trained first aiders (psychological assistants) to accompany and support clients until their condition stabilizes sufficiently for an immediate appointment with a psychologist. Common crisis conditions include self-harming behaviors, suicidal ideation, and suicide attempts.

#### 3.1.2 Complex Clinical Background Underlying Crisis Situation

Participants also emphasized that crises are often embedded in complex psychological and social contexts. Many clients presenting in crisis had underlying mental health issues such as depression, anxiety, bipolar disorder, and borderline personality disorder. Assessments indicated that clients in crisis often had a prior psychiatric history and/or had previously accessed mental health professionals regularly. Issues related to gender identity, such as LGBT and non-binary identities, were also present among clients.

### 3.2 Navigating Crisis Assessment in Complex Situations

#### 3.2.1 Assessing Suicide Risk and Crisis Severity

The first step participants take is to assess the client’s crisis condition. This assessment focuses on the presence of self-harm or suicidal intent. When such intent is identified, participants further explore the intensity, duration, and frequency of these thoughts or urges. Participants also identify the specific forms of self-harm or suicide attempts and examine the factors contributing to the client’s crisis state. The importance of this assessment was reflected in participants’ descriptions of their clinical approach:

“We assess whether there is suicidal intent. If so, how intense it is, how frequently it occurs, how strong the urge is, and whether there is already a plan. We also assess self-harm behaviors, including their frequency, duration, and intensity” (P4).

#### 3.2.2 Exploring Client’s Psychological and Social Context

Participants conduct an in-depth exploration of clients’ conditions and needs by assessing their thoughts, emotions, and physical states. Participants also identify clients’ sources of social support, including significant others who are aware of the clients’ difficulties. This process is essential to enable participants to involve appropriate individuals when necessary. As reflected in participants’ accounts: “I usually explore how the client relates to their family and whether the social support they have is sufficient, or what additional support we can provide as a crisis service. For example, what kind of social support might be needed?” (P3). In addition, to optimize the crisis counseling process, participants assess coping strategies previously used by clients and evaluate them collaboratively. Participants also explore clients’ expectations regarding the outcomes of the counseling sessions.

“We also assess what the client has already tried. In some cases, they may have used self-help strategies that were ineffective. Through this assessment, we can offer alternative perspectives or strategies that they have not previously tried. Sometimes clients say, ‘I have tried this, and it did not work, so why is the psychologist suggesting it again?’ That is why this assessment is necessary as part of a standardized psychological evaluation ” (P5).

### 3.3 Professional Responses in Crisis Intervention

#### 3.3.1 Professional Attitudes Guiding Crisis Intervention

When providing crisis intervention, participants are required to act promptly. For clients in severe crises that pose a risk to themselves or others, participants adopt a directive stance. This involves taking firm, unidirectional actions that do not always align with clients’ immediate preferences. Such measures are implemented as risk-mitigation strategies to ensure clients remain safe and do not endanger themselves or others.

“…at that moment, I gave them two options: ‘I inform your parents, or we go to the hospital.’ I said this because it was clear that they needed to be taken to the hospital. When they refused, I told them there were only two options. I then coordinated with the emergency team and immediately contacted them to ask how we could bring the client to the hospital” (P3).

Subsequently, each crisis client is allocated a quota of four counseling sessions. In accordance with this regulation, participants seek to optimize each session. The objective is to ensure that, upon completing the sessions, clients become empowered individuals. The number of sessions utilized depends on the client’s condition and needs. However, several participants recommend that clients reserve one counseling session so that, if they experience a future crisis, emergency services can be accessed again. As one participant explained: “Usually, for clients who really need more support, I use all four sessions. But for those who are already stable, I typically use three and ask them to save one for emergencies” (P3)

#### 3.3.2 Strategies Used to Stabilize and Support Clients

##### 3.3.2.1 Psychological Intervention

In crisis services, the initial psychological intervention provided is Psychological First Aid (PFA), which aims to stabilize the client’s physical and emotional condition. Once the client has reached a stable state, the intervention focuses on client empowerment. Participants invite clients to map the emotional regulation strategies they already possess and to evaluate their effectiveness. If clients already have effective emotional regulation strategies, participants encourage them to continue practicing these strategies. However, if the evaluation indicates that clients do not yet have effective emotional regulation strategies, participants accompany clients in identifying and implementing new strategies. In subsequent sessions, participants then evaluate the effectiveness of these newly applied strategies.

Various forms of emotional regulation are taught. One strategy introduced to clients is journaling. The technical form of journaling may vary according to the client’s condition. If clients are unable to write, they may use voice recordings instead. The purpose of this strategy is to help clients express and release their emotions.

In addition to journaling, grounding or self-soothing techniques are also taught. These techniques aim to help clients feel calmer by utilizing the five senses. As participants described: “… we also have grounding techniques, anchoring a sense of safety through the senses. For example, which sense is strongest-smell, touch, or hearing? If smell, maybe aromatherapy; if touch, a doll or object; if hearing, certain music” (P5). Clients are encouraged to try and identify the strategies they find most effective: “I ask them to explore and experiment on their own to find which strategy works best for them” (P4).

Subsequently, participants also invite clients to develop a safety plan. A safety plan is provided once the client is in a stable condition. The contents of the safety plan include actions the client can take when experiencing psychological discomfort again, an agreement not to engage in self-harm, and a mapping of contacts that can be reached when the client is in crisis.

In some cases, crisis episodes are linked to clients’ non-adherence to psychiatric medication. When this is identified during joint evaluations, participants provide psychoeducation regarding the importance of taking medication regularly. As one participant noted, “For example, if the client has already been diagnosed and the crisis occurs because they stopped taking their medication, then the focus is more on psychoeducation about why medication adherence is important” (P6).

##### 3.3.2.2 Coordinating and Connecting Clients with Support Systems

In cases involving clients in crisis, powerless, and at very high risk, participants coordinate with emergency service managers to bring them to the emergency department at the hospital. In addition, participants coordinate with other parties involved in the client so they understand the client’s condition and can assist with monitoring. Coordination is conducted with the client’s consent. As reflected in participants’ accounts, “I coordinated with the assistant by immediately messaging them, asking how the client could be taken to the hospital” (P3). In situations where clients lived in communal residences, such as dormitories, collaboration with staff was considered essential to ensure safety. One example involved working with a dormitory manager to prevent recurrence of self-harming behavior: “In some cases, I communicated with the dormitory manager because the client lived in a dormitory. To prevent another overdose, I asked that the medication be kept by the security staff” (P1).

For crisis cases in which clients are still functioning, participants invite clients to identify individuals who can serve as sources of support. Participants then encourage clients to contact these individuals. If clients initially refuse, participants gradually persuade them to connect with their support resources. As described in participant narratives: “What I usually do is ask about their situation, who can help them, and who they need help from, and then I persuade them to contact that person themselves” (P2).

##### 3.3.2.3 Referral to Ongoing Professional Services

In handling clients in crisis, participants require collaboration with other professionals. At the end of the fourth session, if participants determine that clients still require ongoing counseling, they provide several recommendations for psychological services that clients can access, such as clinics, community health centers, hospitals, or private practices. Furthermore, if necessary, participants also persuade clients to access psychiatric services. Participants described this referral process as part of an extended care strategy. Another account emphasized the need for timely referral if progress was limited: “If by the second or third session the client has not improved, then a referral to a psychiatrist may be necessary, as pharmacotherapy may be required (P4).

### 3.4 Challenges in Managing Crisis Situations

#### 3.4.1 Diverging Perceptions of Crisis Situation

In providing mental health emergency services, participants sometimes encounter cases that they do not perceive as emergencies or crises. This condition highlights potential differences in how participants and clients understand what constitutes a crisis. Participants generally define a crisis based on the presence or absence of thoughts or behaviors related to self-harm or harm to others and/or suicidal ideation. In contrast, clients may perceive distressing everyday experiences-such as difficulties in completing a thesis, feeling left behind by peers who have graduated, or experiencing discomfort with oneself-as crisis conditions. From a psychological perspective, such experiences are considered psychological problems but do not necessarily meet the criteria for a crisis. Moreover, differences in perceptions of crisis may also occur among psychologists themselves. Participants acknowledged the lack of a shared understanding of the term “crisis”: “The meaning of ‘crisis’ itself may not yet be uniform among psychologists” (P5). Others described how clients’ subjective interpretations often diverge from clinical criteria:

“There are cases that are truly crises, but there are also cases that, after being explored more deeply, turn out to involve psychological problems, but not to the level of a crisis. However, after understanding the client’s perspective, it becomes clear that people have different interpretations of what constitutes an emergency. For example, a client who feels stuck and unable to work on their thesis may consider that a crisis” (P4).

These differences in perception pose distinct challenges for the services participants provide. Variations in perceptions among psychologists may lead some practitioners to manage cases for prolonged periods, even after the client’s crisis has resolved. Concerns were raised about the implications of delaying case closure for clients who no longer required crisis-level support:

“This has been discussed with other psychologists as well. Sometimes it is puzzling why some psychologists are hesitant to close a case. The indicators of a crisis are actually quite clear. Once the crisis has passed, the client should be referred to general services. However, some still insist that the client must continue to be monitored and followed up. As a result, crisis slots are used for clients who are already calmer and should be in general services, while those slots could be allocated to others who truly need them” (P5).

In addition, differing perceptions of crisis between psychologists and clients often lead to unexpected situations. For instance, when participants consider a client to be in an urgent crisis requiring immediate attention, the client may arrive late or prioritize other activities before attending the session. As one participant recounted: “We were waiting, assuming the client needed immediate help at that moment, but they did not come. We could not even reach them. Although we assessed the situation as critical, the client appeared relatively calm” (P6).

#### 3.4.2 Client-related Barriers in Crisis Management

##### 3.4.2.1 Fluctuating Client’s Condition

In managing crisis cases, participants encounter several challenges related to clients’ conditions. In some instances, clients’ conditions deteriorate or worsen during counseling sessions or after several sessions. Such escalation was described in participants’ accounts: “Initially, the client came in calm and appeared stable. However, during the counseling session, when discussing their feelings, they suddenly became hysterical, saying, ‘I want to die; I’m exhausted; please let me die” (P3). Additionally, some clients threaten to end their lives during counseling sessions. One such case was noted: “I once had a client who threatened to use insecticide. They had prepared themselves and brought it into the counseling room, and their desire to end their life was extremely strong” (P1). Such situations require participants to refer clients to advanced services to ensure comprehensive care: “Eventually, I referred them to a psychiatrist for pharmacotherapy” (P2).

##### 3.4.2.2 Client Resistance to Psychological Services

Another challenge participants face is client resistance to the services provided. Clients may be reluctant to be open about their condition due to concerns that their information might be shared with others. As a result, they may not provide a complete picture of their condition during screening, making it difficult for participants to accurately assess the level of crisis: “I had a client from Faculty X. I did not mention that I was also from Faculty X, because in previous experiences, once I mentioned it, the client immediately stopped. I concluded that they were afraid their information would be accessed” (P2).

Furthermore, some participants encounter clients with a background in psychology who tend to analyze the psychologist using theoretical frameworks learned during their studies. This behavior can reduce the client’s focus on the session content and limit the insights gained. As noted in participants’ reflections: “When the client has a background in psychology, there are two possibilities. During assessment, they may analyze the psychologist, and if the psychologist does not align with the theories they learned, the client becomes resistant” (P5). Another added, “Some clients bring their own theoretical frameworks, so they are no longer a ‘blank slate.’ Many end up analyzing the psychologist and comparing the approach with the theories they already have” (P6).

Additionally, clients may refuse referrals to advanced services, such as psychiatric or hospital care, due to the stigma associated with these services. Participants expressed concern that important follow-up care was sometimes delayed or even denied altogether. This has the potential to undermine the effectiveness of the crisis interventions that have been carried out.

The clients’ resistance is also reflected in their low level of commitment. Clients failed to practice emotional regulation strategies taught during sessions, rescheduled frequently, or failed to return for counseling at the agreed-upon times. Some participants suspect that this may be due to the services being provided free of charge, which reduces clients’ willingness to commit. Moreover, some clients register for or use crisis services merely to try them while they are free, even if they are not in a crisis. As reflected in participants’ experience, “In my view, this is what makes emergency services less well-targeted, because people focus on the fact that it is free rather than on its purpose. As a result, commitment declines significantly. Once people hear the word ‘free,’ it becomes difficult” (P6).

##### 3.4.2.3 Limited Social Support System

Another challenge identified by participants is the limited social support available to clients. In some cases, clients do not want others to know their condition and therefore refuse to provide emergency contact information that could be reached in a crisis. Besides that, some clients also have problems or a lack of support from their surroundings. As the participant noted:

“I realized that many clients do not have a support system. Some have spouses, but the spouses refuse to come. In one case, the repeated crises were actually triggered by conflict with the spouse at home. In another case, it involved friends, and the parents rejected the client. How, then, are they expected to cope outside of crisis services?” (P6).

#### 3.4.3 Professional Challenges Faced by Psychologists

Participants encounter several challenges related to professional practice when managing crisis cases. First, flexibility is required in handling clients. This is because clients present with diverse conditions and types of emergencies, making interventions highly dependent on each client’s condition and progress. This also requires participants to exercise professional judgment in responding to clients’ needs. For example, when referring clients to psychiatrists, participants must consider potential risks, such as the possibility that clients may misuse prescribed medication. Second, participants need persuasive skills to encourage clients to consistently access follow-up services, such as psychiatric care or ongoing counseling. Clients may refuse referrals due to the persistent negative stigma associated with mental health services. Third, limited time availability presents a challenge. Although the service operates as an emergency service, participants cannot remain on standby 24 hours a day. Moreover, despite these time constraints, some clients fail to attend sessions as agreed, even when participants are available to provide services.

“I have already persuaded the client to see a psychiatrist, but they refused because they come from a family that understands medications. They kept saying, ‘I already know about medications,’ and then said, ‘If I take medication, that means I’m really sick, right?’ They were still in denial” (P3).

“… Actually, it had been some time since I wanted to refer the client to a psychiatrist. However, because the client knows medications and their functions, I was concerned that an immediate referral might lead to medication misuse. That was something I discussed with the service administrators” (P1).

## 4 DISCUSSION

The findings of this study reveal the complexity involved in understanding and managing psychological crisis cases. The study demonstrates a wide range of crisis cases, including self-harm, suicide attempts, and cases involving emotional escalation during counseling sessions. In terms of assessment and intervention, psychologists applied a systematic approach that prioritized emotional stabilization and client empowerment through safety planning and emotional regulation strategies. This study also identified several challenges, including differences in perceptions of crisis conditions between psychologists and clients, client resistance, limited social support, and low client commitment to the counseling process.

The variety of crisis cases identified in this study, such as self-harm and suicide attempts, indicates a high demand for mental health emergency services. In Indonesia, where the mental health workforce is limited and distribution is uneven [25], university-based services often serve as the primary entry point for students in acute distress. As a result, these services often receive a wide range of crisis presentations. The observed frequency of emotional escalation during counseling suggests that practitioners require advanced clinical skills to navigate unpredictable emotional dynamics, reinforcing the need for continuous professional development in crisis-specific competencies. The alignment of these competencies can contribute to enhancing therapeutic outcomes [31].

The assessment practices employed by psychologists in this study underscore the importance of identifying the intensity, duration, and frequency of suicidal ideation. In resource-constrained environments, such systematic evaluations are critical for triaging cases and determining intervention urgency, aligning with global standards for emergency mental health care [32]. The study also highlights the importance of exploring clients’ social support systems to ensure that interventions can be implemented effectively. Social support from family members, peers, or other significant individuals can play a crucial role in the recovery process by providing emotional reassurance, practical assistance, and encouragement to engage in treatment [33-35]. Previous research suggests that social support plays a protective role by reducing symptom severity and lowering the risk of relapse [36, 37]. Understanding the availability and quality of such support allows practitioners to identify potential resources that may facilitate recovery, as well as possible barriers that could hinder the implementation of intervention strategies. Furthermore, actively involving clients throughout the assessment and decision-making processes can enhance their engagement, compliance, and sense of ownership over the recovery process [38, 39].

Crisis management in this study involved direct interventions such as Psychological First Aid (PFA), the development of safety plans, and the strengthening of clients’ coping strategies. Techniques such as journaling and grounding were found to be effective in helping clients regulate their emotions [40-45]. Emotional stabilization is a critical initial step before designing long-term interventions.

The crisis interventions implemented by psychologists in this mental health emergency service adopted an empowerment-based approach. The development of safety plans, along with evaluation and psychoeducation regarding effective coping strategies, played a crucial role in enhancing resilience and strengthening clients’ capacity to manage crises. This approach not only supports clients in coping with acute situations but also equips them with skills to prevent future crises. Individual empowerment is central to sustainable psychological interventions [46].

Beyond direct client interventions, coordination among psychologists and other parties, such as administrative staff or emergency medical service providers, was vital to crisis management. This collaborative approach reflects the broader structure of mental health service delivery, in which crisis intervention often relies on coordination across multiple sectors. In settings with limited mental health resources, such inter-agency collaboration can help bridge service gaps and accelerate access to appropriate care [47-50]. In highly critical cases, this study found that rapid coordination with medical services can be life-saving.

Several challenges were identified in the development and implementation of crisis services. A fundamental challenge was the differing understandings of crisis conditions between psychologists and clients, as well as among psychologists themselves. Understanding what constitutes a crisis is an essential element of mental health emergency services. This study identified a clear discrepancy between psychologists and clients in defining crisis conditions. Psychologists tend to define crises based on immediate threats, such as suicidal thoughts or behaviors, whereas clients often perceive everyday difficulties, such as problems completing a thesis, as crises. In addition, psychologists’ understanding of crisis conditions was not always consistent. These differences indicate a need for improved education regarding the definition of psychological crises. Individuals’ understanding of mental health conditions is often shaped by subjective perceptions, which can hinder access to appropriate services [51, 52]. Therefore, educational approaches may serve as a strategic means of aligning understanding between psychologists and clients regarding crisis conditions [53].

Other challenges identified in mental health emergency services include client resistance, limited social support, and low client commitment. Client resistance often stems from stigma surrounding mental health services or skepticism regarding the effectiveness of interventions [54, 55]. Specialized training for psychologists can help address these barriers [56]. In addition, limited social support frequently exacerbates clients’ conditions, particularly among those lacking emotional or financial resources. In this context, crisis services need to integrate community-based support to strengthen intervention outcomes.

This study has several limitations. This study was conducted at a single university, resulting in a relatively small and homogeneous sample that may not reflect the diversity of higher education institutions in Indonesia. The focus on offline services excludes the burgeoning field of digital or tele-mental health support. Additionally, the focus group format may have introduced social desirability bias, where participants align their responses with professional norms. Moreover, although the facilitator was a psychologist who was not directly involved in crisis intervention services, the shared professional background between the facilitator and participants may have influ-enced the interaction and responses during the discussion.

This study offers important implications for the development of mental health emergency services, particularly in aligning the conceptualization of “crisis” between psychologists and clients. The findings highlight the need for broader public education about psychological crises so that individuals can access services that align with their needs. Furthermore, the diverse nature of encountered cases necessitates continuous professional training in empowerment-based interventions, specifically Psychological First Aid and safety planning. Additionally, findings on client resistance and limited social support underscore the importance of inter-agency collaboration and the integration of community-based services to enhance the effectiveness of mental health emergency care. This study also reinforces the need for a holistic mental health service system. Integrating community support, technology, and professional training is key to improving service effectiveness. Collaborative approaches can improve service outcomes and expand access for clients [47-50]. Therefore, mental health emergency services must continue to adapt to evolving societal needs, including addressing persistent stigma and systemic barriers.

For practitioners, these findings emphasize that a personalized approach must extend beyond the counseling room through the active integration of social support systems. By framing social support as a vital extension of clinical care, practitioners can better ensure post-intervention stability once formal sessions conclude. Technological innovations increasingly facilitate this holistic model; specifically, digital health platforms bridge accessibility gaps for underserved populations, allowing for more consistent engagement with support networks [57]. Furthermore, the inclusion of artificial intelligence optimizes this ecosystem by refining risk prediction and supporting clinical decision-making [58]. Collectively, these elements provide a practice-based foundation for developing responsive, inclusive, and evidence-based mental health policies within higher education.

## CONCLUSION

This study reveals that mental health emergency services face significant challenges in understanding and managing the crises experienced by clients. Differences in perceptions between clients and psychologists regarding the definition of a crisis constitute one of the primary barriers to effective service delivery. While clinical management-utilizing Psychological First Aid (PFA), safety planning, and emotional regulation-provides a systematic response, its effectiveness is often hindered by client resistance, limited social support, and fluctuating commitment. To address these barriers, cross-sector collaboration and comprehensive public education are required. This study underscores the need to develop a holistic, technology-based mental health emergency service system that prioritizes client empowerment.

## RECOMMENDATIONS

Future studies should evaluate the efficacy of technology-driven interventions, such as telemedicine and AI-supported risk prediction, within the Indonesian cultural context. Further exploration of crisis management across different age groups is also necessary, as each developmental stage presents distinct characteristics and intervention needs. Additional studies are needed to evaluate the long-term impacts of crisis interventions, such as safety plans and emotional regulation strategies, on clients’ ability to manage crises independently. Moreover, future research should examine the relationship between social support and intervention outcomes, as this study's findings indicate that limited social support often hinders client recovery.

For psychological practitioners, it is essential to continuously enhance professional competencies through intensive training in crisis management, particularly in situations involving emotional escalation and psychological complexity. Practitioners are also encouraged to strengthen collaboration with other stakeholders, such as emergency medical services, local communities, and clients’ families, to ensure continuity of support after counseling sessions conclude. In addition, public education efforts should be intensified to improve understanding of psychological crisis definitions and to reduce stigma toward mental health services. By integrating technology into service delivery, practitioners can expand accessibility and ensure that clients in remote areas or those with physical limitations can receive optimal mental health emergency services.

## Figures and Tables

**Table 1:** Themes and subthemes.

**Themes**	**Subthemes**
Understanding and interpreting crisis situation	Acute crisis presentations requiring immediate response
	Complex clinical background underlying crisis situation
Navigating crisis assessment in complex situation	Assessing suicide risk and crisis severity
	Exploring client’s psychological and social context
Professional responses in crisis intervention	Professional attitudes guiding crisis intervention
	Strategies used to stabilize and support clients
Challenges in managing crisis situations	Diverging perceptions of crisis situation
	Client-related barriers in crisis management
	Professional challenges faced by psychologist

## Data Availability

The data are available from the corresponding author [A.S.L.R] upon reasonable request and subject to ethical approval.

## LIST OF ABBREVIATIONS

FGD: Focus Group Discussions
LGBT: Lesbian, Gay, Bisexual, Transgender
PFA: Psychological First Aid

## STANDARDS OF REPORTING

COREQ guidelines were followed.
